# RhoE Promotes Metastasis in Gastric Cancer through a Mechanism Dependent on Enhanced Expression of CXCR4

**DOI:** 10.1371/journal.pone.0081709

**Published:** 2013-11-29

**Authors:** Bin Feng, Kai Li, Haixing Zhong, Gui Ren, Hefei Wang, Yulong Shang, Ming Bai, Jie Liang, Xin Wang, Daiming Fan

**Affiliations:** 1 State Key Laboratory of Cancer Biology and Institute of Digestive Diseases, Xijing Hospital, The Fourth Military Medical University, Xi’an, Shaanxi, China; 2 Department of Anesthesiology, Xijing Hospital, The Fourth Military Medical University, Xi’an, Shaanxi, China; University of Kansas Medical Center, United States of America

## Abstract

RhoE, a novel member of the Rho protein family, is a key regulator of the cytoskeleton and cell migration. Our group has previously shown that RhoE as a direct target for HIF-1α and mediates hypoxia-induced epithelial to mesenchymal transition in gastric cancer cells. Therefore, we assumed that RhoE might play an important role in gastric cancer metastasis. In the present study, we have explored the role of RhoE expression in gastric cancer, cell invasion and metastasis, and the influence of RhoE on regulating the potential expression of down-stream genes. RhoE expression was elevated in gastric cancer tissues as compared with normal gastric tissues. We also found a close correlation between the histological grade and the diagnosis of the patient. Up-regulation of RhoE significantly enhanced the migratory and invasive abilities of gastric cancer cells both *in vitro* and *in vivo*. Moreover, down-regulation of RhoE diminished the metastatic potential of cancer cells. PCR array and subsequent transwell assay showed that the regulation of gastric cancer metastasis by RhoE was partially mediated by CXCR4. This observation suggested that CXCR4 might be a downstream effector for RhoE. In summary, our study identified RhoE as a novel prognostic biomarker and metastatic-promoting gene of gastric cancer.

## Introduction

Gastric cancer is the second leading cause of cancer-related death in the world and tumor metastasis is the biggest obstacle to its successful treatment and the major cause of patient mortality[1,2]. For decades, numerous studies have attempted to understand the processes and mechanisms of metastasis in gastric cancer. In recent years, it has been found that epithelial mesenchymal transition (EMT) is of crucial importance in this process and greatly contributes to cancer cell invasion and metastasis. One possible reason is that during EMT, the cytoskeleton is fundamentally modulated, leading to the scattering of tumor cells[3].

RhoE belongs to a sub-group of the Rho proteins, which play key roles in cytoskeleton formation, cell motility, cell cycle and apoptosis[4,5]. Unlike the typical Rho proteins, which cycle between an active GTP-bound state and a resting GDP-bound state, RhoE lacks intrinsic GTPase activity and always presents as the active GTP-bound form[6]. This unique feature indicates that the function of RhoE is essentially regulated through its expression rather than by its activity. 

Recently, several studies found that abnormal expression of RhoE was associated with carcinogenesis and that RhoE might act either as a tumor suppressor gene or an oncogene, depending on the origin of the cancer[7-9]. Similarly, RhoE has varying influences on metastasis in different cancers. In melanoma, RhoE supports the migration and invasiveness of tumor cells by regulating the actin cytoskeleton[10]. However, in hepatocellular carcinoma, RhoE represents itself as a suppressor protein of tumor metastasis[11,12].

Previously, our group has found that RhoE was enhanced in gastric cancer cells by induction of HIF-1α expression under hypoxic conditions and promoted EMT of gastric cancer cells[13]. Thus, we hypothesized that RhoE may have a positive contribution in the metastasis of gastric cancer. In the present study, we investigated the affect of RhoE on the metastasis of gastric cancer cells and the underlying mechanisms.

## Materials and Methods

### Cell Culture

The human gastric adenocarcinoma cell-lines SGC7901, MKN-45, MKN-28, KATO-III and the normal gastric mucosal cell-line GES were preserved in our institute[14-16]. Additionally, the gastric cancer cell- line SGC7901-M, which has a high metastatic potential, and SGC7901-NM, which has a poor metastatic ability, were established and maintained in our institute[17]. All the cells were maintained in RPMI 1640 medium supplemented with 10% fetal bovine serum (GIBCO, Carlsbad, CA, USA) and cultured at 37°C in a fully humidified atmosphere of 5% CO2. 

### Tissue Array

For immunostaining applications, two tissue arrays of human gastric cancer were commercially purchased from Outdo Biotech Co. Ltd (Shanghai, China). One tissue array contained 90 tumor tissue spots and matched adjacent non-tumor tissue spots that were obtained from 90 patients (with 5.3-6.1 years follow-up). The manufacturer provided the gender, age, and clinicopathological parameters of the patients. The other array contained 120 spots with 40 primary tumor tissue spots, 40 matched adjacent non-tumor tissue spots, and 40 matched lymph node metastases spots.

### Immunohistochemistry

Immunohistochemistry was performed using Histostain™ Plus kits (Zhongshan Goldenbridge Biotech, China) according to the manufacturer’s instructions. The mouse anti-RhoE antibody (diluted 1:100; Upstate, MA, USA) or anti-CXCR4 antibody (diluted 1:200; Sigma-Aldrich, MO, USA) were used in the IHC assay as first antibody. Mouse IgG was used instead of the first antibody as the negative control in these studies. The obtained result was semi-quantitatively evaluated by assigning scores for the intensity of immunoreactivity and for the proportion of cells positively stained as described by others. The intensity of immunoreactivity was divided into four categories and scored as absent (–; score: 0), weak (+; score: 1), moderate (++; score: 2), or strong (+++; score: 3) according to the staining intensity that was observed in the majority of gastric epithelial cells. The proportion of positive cells was classified into four groups: (1), 0–25% of tumor cells exhibiting immunoreactivity; (2), 25–50% of cells; (3), 50–75 percentage points of cells; and (4), 75–100% of cells. The overall score was the product of the two [18].

### Western Blot Analysis

Protein samples were extracted from cells or tissues by sonication in a lysis buffer (150 mM NaCl, 50 mM Tris–HCl (pH8.8), 0.1% SDS, 2 mM EDTA, 1 mM PMSF, 1% NP40, 5 μg /ml aprotinin, 1 μg /ml leupeptin) on ice, then centrifuged at 12,000 rpm for 10 min. Proteins were resolved by SDS-PAGE and electrotransferred to nitrocellulose membranes (Bio-Rad, CA, USA). Non-specific binding was blocked with 5% fat-free milk for 1 hour at room temperature. The membrane was then separately incubated with anti-RhoE antibody (diluted 1:400; Upstate, MA, USA), anti-CXCR4 antibody (diluted 1:1000; Sigma-Aldrich, MO, USA), anti-VEGFA antibody (diluted 1:500; Abcam, MA, USA), anti-CD82 antibody (diluted 1:1000; Abcam, MA, USA), anti-CTSK antibody (diluted 1:1000; Santa Cruz, Texas, USA) or β-actin antibody (diluted 1:10000; Sigma-Aldrich, MO, USA) overnight at 4°C. After four washes with TBS-T buffer, the membranes were incubated with the horseradish peroxidase-conjugated secondary antibody (diluted 1:2000; Santa Cruz, Texas, USA) for 1 h at room temperature. After 4 washes with TBS-T buffer, the bands were developed with SuperSignal Chemiluminescent Substrate (Thermo Scientific, MA, USA) to visualize the proteins. Blots were then analyzed by Quantity One® software (Version 4.2. Bio-Rad, CA, USA) following the User’s Guide. Western blot for β-actin expression was used as an internal control.

### Ectopic Expression and siRNA-Mediated Silencing

For ectopic expression of target genes, gastric cancer cells were transduced with lentivirus vector expressing RhoE or CXCR4 (provided by GeneChem, Shanghai, China). Briefly, cells were plated and grown to 75 to 80% confluence without antibiotics, following which the lentiviral vectors were added to the cells in growth medium containing polybrene (2 μg/ml) at an MOI of 50. Next, cells were cultured for another 72 h and the infection efficiency was checked by fluorescence microscopy and Western blot analysis. Interference of human RhoE or CXCR4 expression was achieved using an RNA interference technique. siRNA duplexes were synthesized by Takara Biotechnology (Liaoning, China) and the targeted sequences of RhoE or CXCR4 were defined thus:

RhoE, 5’-GAACGUGAAAUGCAAGAUAUU-3’;
CXCR4, 5’-UAAAAUCUUCCUGCCCACC-3’.

Control siRNA duplexes were designed after interrogating the BLAST database with non-specific targeting sequences. Cells were transfected with oligonucleotide duplexes using LipofectamineTM 2000 (Invitrogen, NY, USA) according to the manufacturer’s instructions, following which they were subjected to further analyses 48 h post-transfection. 

### Wound-Healing Assay

To detect cell motility, exponential phase cells were seeded into 6-well plates. After the cells reached confluence as a monolayer of cells on the plate substrate, a plastic pipette tip was drawn across the center of the plate to produce a clean 1 mm wide wound area and the medium was replaced with fresh RPMI1640 containing 1% fetal bovine serum. After 48 h, cell movement into the wound area was assessed using a phase-contrast microscope as previously described[19,20].

### Invasion Assay

Cell invasion assays were performed using a transwell plate (Corning, NY, USA) coated with Matrigel (Becton Dickinson, NJ, USA). Briefly, Matrigel was diluted to a concentration of 2 mg/ml, and 50 μl of this solution was placed into a polycarbonate filter and air-dried. After rinsing with PBS, the filters were placed into wells and 700 μl of RPMI-1640 culture medium supplemented with 10% FBS was added into the lower chamber. Cells were resuspended in RPMI-1640 containing 1% FBS and 1×105 cells in 0.2 ml defined medium were plated into the upper chamber. Cells in the invasion chambers were incubated in a humidified incubator for 12–48 h. After an additional incubation, cells that penetrated the pores of the membrane and dispersed to the lower surface of the filters were stained with 5% crystal violet solution for visualization. Next, the invaded cells in each well were counted under a light microscope (Olympus, Tokyo, Japan) and quantified from visualizing five random fields at a magnification of × 200 and averaged as described by Albini[21]. The invasion assay was repeated 3 times. The value of the bar graph represents the average number of invaded cells from 3 separate experiments.

### Tail Vein Metastatic Assay

The tail vein metastatic assay was determined as previously reported[22]. Thirty male nude mice were handled using best humane practices and were cared for in accordance with NIH Animal Care and Use Committee guidelines. Cells were harvested using trypsin and washed three times with PBS. Mice were then injected with 1 × 106 cells in 0.1 mL PBS through tail vein injection. The mice were then monitored for overall health and total body weight. On the 28th day after injection, the mice were sacrificed. Lung and liver tissues were observed by naked eye and the number of visible tumors on the lung and liver surface were quantified. Lung and liver tissues were cut into serial sections, stained with hematoxylin and eosin, then observed under a light microscope. Experimental and control groups each contained 6 mice. All experimental procedures were approved by the Experimental Animal Welfare and Ethics Committee, the Fourth Military Medical University. Animal experiments were performed with the approval of the Institutional Committee for Animal Research, in conformity with national guidelines for the care and use of laboratory animals.

### Tumor Metastasis PCR Array

The expression of metastasis-associated genes were determined by the Human Tumor Metastasis PCR Array (PAHS-028A; SuperArray Biosciences, Maryland, USA) according to the manufacture’s instructions. First, total RNA was extracted from RhoE-lentviral infected SGC7901 cells and control cells using standard protocols, which was then converted to first-strand cDNA. The cDNA template was combined with a 2×SuperArray PCR master mix, added to the wells of a PCR Array plate (384-well) containing the gene-specific primer sets, and subjected to real-time PCR. The PCR cycling conditions were as follows: 40 cycles at 95°C for 15 s, 60°C for 1 min, and 72°C for 30 s. Five housekeeping genes were used as internal controls. The fold-change in the expression of metastasis-related genes was determined by the ΔΔCt method.

### Statistical Analysis

Data analysis was performed by using the SPSS 17.0 statistical analysis software (Chicago, IL, USA). The Mann-Whitney U test was used to evaluate the significance of differences in the frequency of expression of RhoE between gastric cancer tissues and lymph node metastases. The Kruskal-Wallis H test for multi-groups, and the Mann-Whitney U test for two group comparisons were employed to analyze the relationship between both RhoE expression levels and various clinico-pathologic factors of gastric cancer specimens. Cumulative survival time was calculated using the Kaplan–Meier survival method and analyzed by the log-rank test. Univariate and multivariate analyses were based on the Cox proportional hazards regression model. Comparisons of quantitative data were analyzed by Student’s T -test between two groups. Correlation analysis between the expression of RhoE and CXCR4 was estimated by Spearman’s rank correlation method. Differences were considered statistically significant when P <0.05. 

## Results

### Over-expression of RhoE is correlated with the differentiation grade and tumor staging in gastric cancer tissues and indicates a poor prognosis for patients

**Figure 1:**
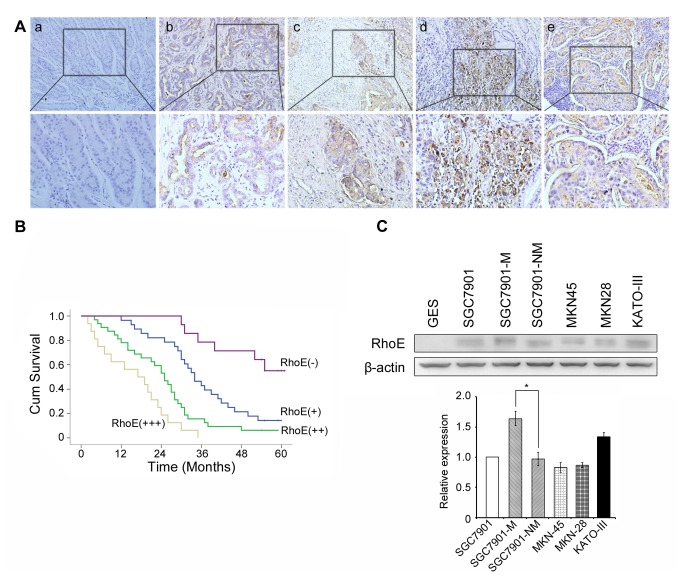
Expression of RhoE in gastric cancer tissues and cells, and its association with patient survival. (**A**), Immunohistochemical analysis of RhoE in tissues; (**a**), normal gastric epithelium; and (**b**) well-differentiated, (**c**) moderately differentiated or (**d**) poorly-differentiated gastric cancer tissue; (**e**) metastatic site in the lymph node. Brown coloration represents positive RhoE staining. (**B**), Kaplan- Meier post-operative survival curve as a function of RhoE expression. (**C**), Western blot analysis of RhoE expression in different gastric cell-lines. β-actin was used as the internal control. The relative expression levels of RhoE in gastric cancer cell-lines were presented as bar charts. * P <0.05.

The expression of RhoE in gastric cancer tissues, adjacent non-tumor tissues and related metastatic lymph node tissues was examined by immunohistochemistry (Figure 1A). RhoE expression was found weakly expressed or even absent in adjacent non-tumor tissues (a), while its expression was significantly higher in gastric cancer tissues and lymph node metastases (b-e). Next, we analyzed the relationship between RhoE staining and the clinicopathological parameters of gastric cancer patients (Table 1). The results showed that neither gender nor age was correlated with the expression of RhoE. However, increased RhoE expression was statistically correlated with the grade of differentiation (P = 0.005) and tumor staging, which consisted of tumor size (T, P <0.001), lymph node invasion (N, P = 0.001), and distant metastasis (M, P = 0.003). Furthermore, Kaplan–Meier survival analysis showed that patients with high expression levels of RhoE, presented with a shorter overall survival as compared those patients with negative expression of RhoE (Figure 1B). Multivariate Cox proportional hazards model assessments indicated that the levels of RhoE expression were an independent and significant factor for survival in gastric cancer patients (Table S1). 

**Table 1:** Statistical Analysis of Immunohistochemical Assay.

		**Expression level of RhoE**				
**Category**	**n**	-	+	++	+++	**P value**
Total	90	14	28	32	16	
**Age**						0.421
**≤60**	41	6	10	18	7	
**>60**	49	8	18	14	9	
**Gender**						0.742
**Male**	67	9	23	22	13	
**Female**	23	5	5	10	3	
**Differentiation**						0.005^a^
**Well**	5	2	1	2	0	
**Moderately**	47	12	15	14	6	
**Poorly**	38	0	12	16	10	
**TNM stage**						
**T**						<0.001^a^
**T1**	9	4	3	2	0	
**T2**	17	5	8	4	0	
**T3**	47	4	14	20	9	
**T4**	17	1	3	6	7	
**N**						0.001^b^
**N0**	31	11	10	7	3	
**N1-N3**	59	3	18	25	13	
**M**						0.003^b^
**M0**	80	14	27	28	11	
**M1**	10	0	1	4	5	

^a^, Kruskal-Wallis H Test; ^b^, Mann-Whitney U Test

Subsequently, we studied the expression levels of RhoE in primary gastric cancer tissues and their matched metastatic lymph node tissues. The expression levels of RhoE were absent in 7 gastric cancer tissues, weakly expressed in 16 gastric cancer patients, moderately expressed in 11, and highly expressed in 6 cancer tissues, respectively. By contrast, in lymph node metastases the corresponding number was 3, 12, 14 and 11. In addition, the expression of RhoE was significantly higher in metastatic lymph node tissues than that found in primary cancer tissues. These observations indicated that RhoE expression was correlated with metastasis in gastric cancer (Table 2, P< 0.05).

**Table 2:** Expression of RhoE in Primary Cancer Tissues and Matched Lymph Node Metastases.

		**Expression level of RhoE**				
**Histological type**	**n**	-	+	++	+++	P Value
**Primary cancer tissues**	40	7	16	11	6	0.048*
**Lymph node metastases**	40	3	12	14	11	

^*^ Mann-Whitney U Test

Expression of RhoE in cell-lines was analyzed by Western blot (Figure 1C). We found that compared to that in the GES cell-line, the expression of RhoE was significantly higher in various gastric cancer cell-lines. Moreover, the level of RhoE protein expression was significantly higher in the highly invasive SGC7901-M cell sub-line than in the less invasive cell sub-line SGC7901-NM. Therefore, further studies of tumor metastasis were done based on cellular modeling with the SGC7901-M and SGC7901-NM cell-lines.

### RhoE promotes the migratory and invasive abilities of gastric cancer cells in vitro and in vivo

**Figure 2:**
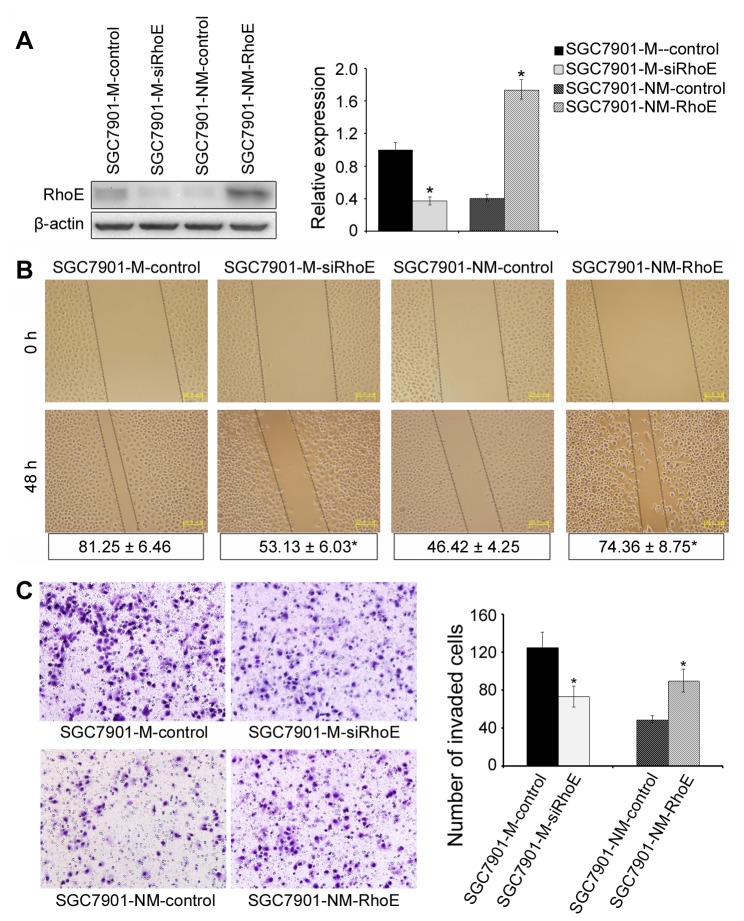
RhoE promotes the migratory and invasive abilities of gastric cancer cells *in* *vitro*. (**A**), RhoE was down-regulated in SGC7901-M cells after treatment with siRNA while RhoE expression was up-regulated in SGC7901-NM cells after treatment with lentivirus. RhoE protein levels were confirmed by Western blot analysis. (**B**), The migratory ability of the cells was evaluated by a wound-healing assay. The wound widths of each sample were measured at different time-points by phase-contrast microscopy (Olympus, Tokyo, Japan), and the closure ratio was calculated in accord with the following formula: Wound Closure (%) = (width 0 h) - width 24 h) / width 0 h. *P <0.05. These results were then compared to those of the control cells. (**C**), Tumor cell invasion activities were measured by transwell assay. Representative image fields of invasive cells on the membrane are shown. Data are represented as normalized cellular invasion (invasion index) relative to control cells. *P <0.05. The images shown are representative of three experiments.

To explore the role of RhoE in gastric cancer metastasis, we up-regulated its protein expression in SGC7901-NM cells following transduction with the RhoE lentivirus vector or control vector (named SGC7901-NM-RhoE or SGC7901-NM-control). By contrast, dampening of RhoE expression in SGC7901-M cells was achieved following transfection with siRNA targeted against RhoE mRNA or with control siRNA (named SGC7901-M-siRhoE or SGC7901-M-control respectively). Protein expression levels were confirmed by Western blot analysis after transfection (Figure 2A).

Next, *in vitro* migration and invasion assay were performed (Figure 2B and 2C). We found enhanced expression of RhoE could significantly up-regulate the migratory and invasive abilities of the gastric cancer cell-line SGC7901-NM, in wound healing and transwell invasion assays. By contrast, SGC7901-M cells, exhibiting dampened expression of RhoE showed a remarkable inhibition of migratory and invasive abilities as compared with control cells. To understand whether the functions of RhoE were common events in various gastric cancer cells or SGC7091-specific events, the gastric cancer cell-lines MKN45 and MKN28 were used to complete the same experiments. The results of both the wound-healing and transwell invasion assays revealed that RhoE promoted the migratory and invasive abilities of both MKN45 and MKN28 cells (Figure S1), which suggested that the functions of RhoE that were detected *in vitro* were comprehensive for all gastric cancer cell-lines. Meanwhile, we also performed MTT assay to test wether alteration of RhoE expression could affect cell growth of gastric cancer in our study. As Figure S3A shown, neither up- regulation of RhoE in SGC7901-NM cells nor down-regulation of RhoE in SGC7901-M cells could cause marked change of cell proliferation (p > 0.05), thus excluded the effect of RhoE on cell proliferation which would bring confusion to our results and further confirmed that RhoE can promote cell motility of gastric cancer cells.

**Figure 3:**
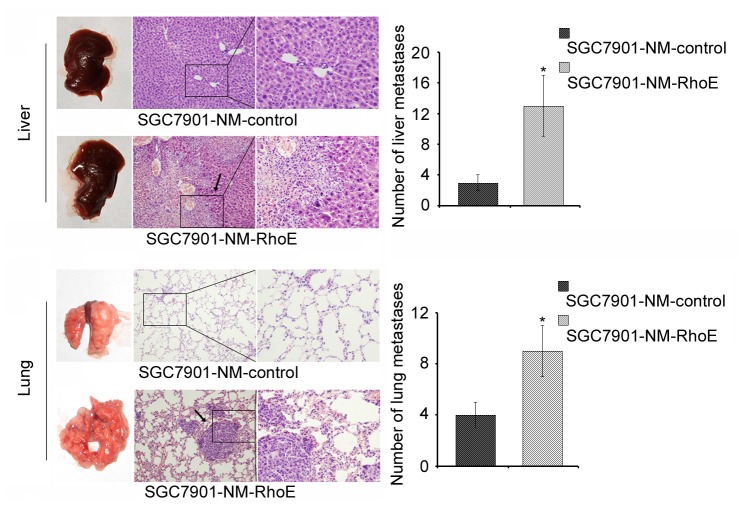
RhoE promotes the metastatic ability of gastric cancer cells *in* *vivo*. H and E staining of both the lungs and livers were assessed from mice that had received intravenous tail injections of SGC7901-NM-RhoE and control cells respectively. Metastatic loci were identified and marked by arrows. The number of metastatic loci in the liver and lung were also counted (middle). *P <0.05.

To further investigate whether increased RhoE could alter the *in vivo* metastatic ability of gastric cancer cells, we performed *in vivo* tail vein metastatic assays in nude mice using SGC7901-NM-RhoE and SGC7901-NM-control cell-lines. In sacrified mice, it was found that cells displaying higher levels of RhoE expression led to significantly more visible liver- and lung-surface tumors as compared with control cells (P <0.05, Figure 3). H and E staining showed that SGC7901-NM-RhoE cells apparently produced metastases in both livers and lungs, while control cells displayed only partial metastases. Taken together, these data suggested that RhoE played an important role in promoting metastasis of gastric cancer cells both *in vitro* and *in vivo*.

### CXCR4 could be a down-stream gene of RhoE in gastric cancer

To determine the possible down-stream genes through which RhoE may mediate its function in the metastasis of gastric cancer cells, we performed PCR Array to explore the differentially expression of metastasis-related genes shared between SGC7901-NM-RhoE and SGC7901-NM-control cell-lines. Genes which increased or decreased ≥ 2 fold were considered as potential RhoE-dependent downstream genes. In sum, we detected 84 genes and ultimately found 6, which were markedly changed after expression of RhoE was enhanced (Table 3). Among the 6 genes, 3 (CXCR4, VEGFA, CTSK) were up-regulated, and three others (MMP7, CD82, CTSL1) were down-regulated in SGC7901-NM-RhoE cells. 

**Table 3:** Differentially Expressed Metastasis-Related Genes after Augmented RhoE Expression.

		**Expression level of mRNA**	
Gene name	**Description**	**SGC7901-NM-control**	**SGC7901-NM-RhoE**
CXCR4	Chemokine (C-X-C motif) receptor 4	1.00	3.30
VEGFA	Vascular endothelial growth factor A	1.00	2.78
CTSK	Cathepsin K	1.00	2.68
CTSL1	Cathepsin L1	1.00	-2.12
CD82	CD82 molecule	1.00	-2.29
MMP7	Matrix metallopeptidase 7	1.00	-2.47

**Figure 4:**
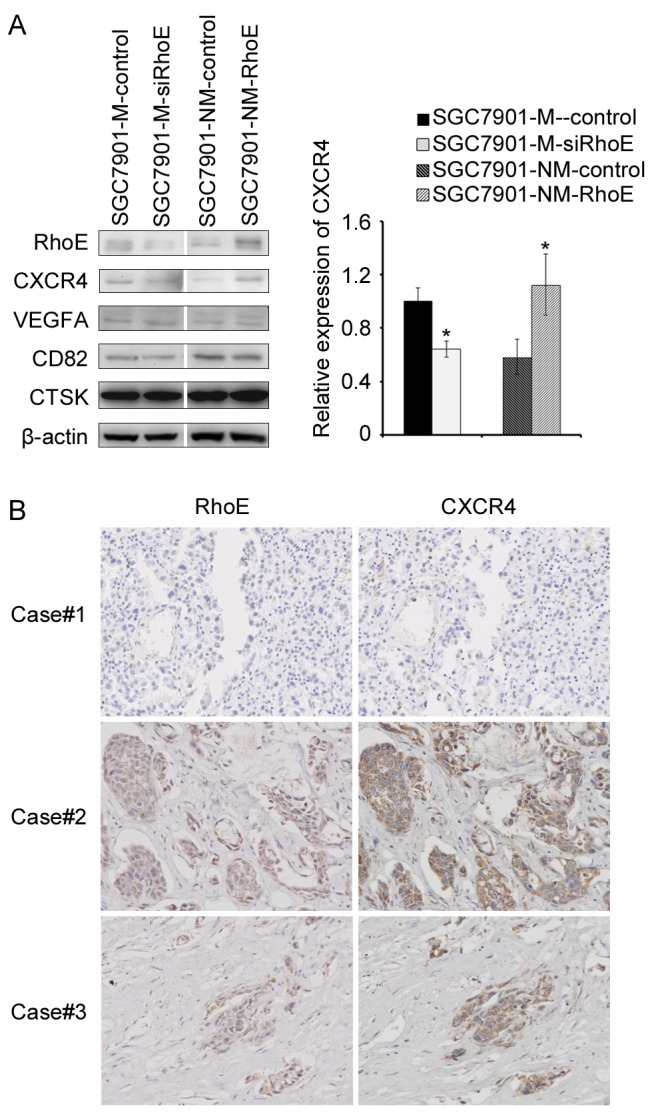
Verification of the expression of downstream genes in different cell-lines by Western blot analysis of RhoE. (**A**) RhoE, CXCR4, VEGFA, CD82 and CTSK expression of SGC7901-M-control, SGC7901-M- siRhoE, SGC7901-NM-control, and SGC7901-NM-RhoE cell-lines. The expression of β-actin was used as internal control. The relative expression levels of CXCR4 were shown as bar charts. *P <0.05. Values represent the arithmetic mean and standard error of the mean (SEM) as determined from at least three experiments. (**B**) Immunohistochemical staining of RhoE and CXCR4 in 60 pairs of gastric cancer tissues. Three representative cases showed that CXCR4 expression was well-correlated with that of RhoE.

In this study, the expression of CXCR4, VEGFA, CTSK and CD82 were verified by Western blot analysis and interestingly, we found that only CXCR4 expression was consistent with the results obtained by PCR array analysis. The protein level of CXCR4 was suppressed by interference of RhoE and by contrast, was increased when RhoE was ectopically expressed (Figure 4A). Thus, the correlation between RhoE and CXCR4 expression was analyzed by immunohistochemistry in 60 gastric cancer tissues The results showed that CXCR4 was highly expressed in tissues where RhoE was positively stained, while it was poorly expressed in those tissues where RhoE was expressed at a low level (Figure 4B). In addition, both RhoE and CXCR4 expression were concordant in 83.3% (50/60) of gastric carcinomas specimens, the Spearman R correlation coefficient was 0.80 (P <0.001) and indicated a close correlation between both RhoE and CXCR4 expression in gastric cancers (Table 4). As reported, over-expressed CXCR4 also plays an important role in cancer metastasis and is involved in the EMT process, which suggested that CXCR4 might be a downstream gene of RhoE with importance in the metastasis of gastric cancer cells[23]. 

**Table 4:** Correlation Between Expression of RhoE and CXCR4 in 60 pairs of Gastric Cancer Tissues.

	**CXCR4**						**Spearman’s correlation**	
**RhoE**	**+**	**++**	+++	**Negative**	**n**	**p**	**p**	**R**
**+**	18	2	0	0	60	<0.001	<0.001	0.80
**++**	1	17	3	0				
+++	0	2	7	0				
**Negative**	2	0	0	8				

### CXCR4 is essential in inducing invasion by RhoE in gastric cancer cells

**Figure 5:**
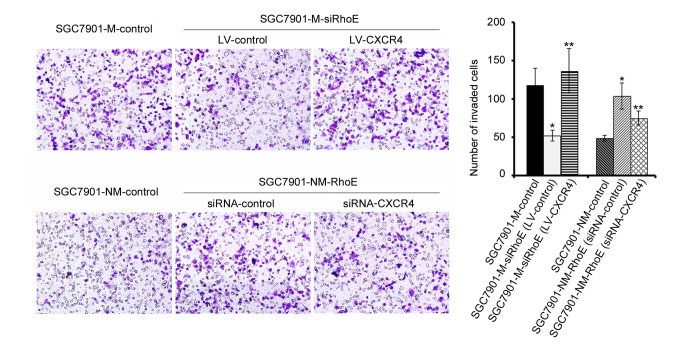
RhoE induced metastasis of gastric cancer cells was mediated by CXCR4. The influence of CXCR4 on RhoE induced metastasis was measured by a transwell assay. In SGC7901-M-siRhoE cells, CXCR4 was enhanced following lentivirus infection. In SGC7901-NM-RhoE cells, CXCR4 was knocked out by treatment with siRNA interference. The number of invading cells was determined and presented as bar charts. *P <0.05, in comparisons between the cell-lines exhibiting different levels of expression of RhoE. **P <0.05, in comparisons between cell-lines displaying differential levels of expression of CXCR4.

To investigate the effect of CXCR4 on the ability of RhoE to function in the metastasis of gastric cancer, we enhanced the expression of CRCR4 in SGC7901-M-siRhoE cells by lentiviral tranduction and down-regulated its expression in SGC7901-NM-RhoE cells by siRNA interference. The protein level was determined by Western blot analysis (Figure S2). In the following transwell assay, we found that enhanced expression of CXCR4 could restore the invasive ability of SGC7901-M-siRhoE cells, while by contrast silencing endogenous CXCR4 repressed the invasion of SGC7901-NM-RhoE cells (Figure 5). These observations indicated that RhoE induced gastric cancer cell invasion, which was partially mediated by CXCR4. These observations also further demonstrated that CXCR4 was a down-stream target of RhoE. 

## Discussion

Aberrantly-expressed RhoE is often associated with tumor progression. However, the role o RhoE may shift between tumor suppressor and oncogene depending on the origin of the tumor[7-9]. As reported, gastric cancer remains one of the most common cancers worldwide, especially in East Asia. Previously, it has been shown that abnormal RhoE expression is well correlated with the progression of gastric cancer. As Zhou and Li described, the expression of RhoE is increased in gastric cancer by the induction of HIF-1α, and whose expression could go augmented still more when cancer cells generate resistance to anti-tumor drugs. In addition, enhanced expression of RhoE promotes drug resistance by suppressing Bax expression[13,24]. 

In the present study, we found that RhoE expression was elevated in gastric cancer tissues whereas it was absent or weakly expressed in adjacent non-tumorous tissues. Additionally, clinical evidence showed that RhoE over-expression was significantly correlated with the degrees of cancer cell differentiation and TNM staging, which consists of tumor size (T), lymph node invasion (N) and metastasis (M). More importantly, RhoE expression levels were an independent and significant risk factor for survival in gastric cancer. Moreover, up-regulated expression of RhoE could predict a poor outcome in gastric cancer patients. Taken together, our data strongly suggested that RhoE might have served as an oncogene in gastric cancer and played a positive role in gastric cancer progression, especially in cancer metastasis. It is formally possible that RhoE could be a novel prognostic factor in patients with gastric cancer. Thus, considering that metastasis is one of the major reasons for gastric cancer mortality, our findings might provide a new clue or new target to inhibit tumor metastasis in gastric cancer and might finally assist in the development of therapeutic strategies to prolong the survival of patients.

Nevertheless, the role of RhoE in cancer metastasis remains partially undetermined. In hepatocellular carcinoma and mesenchymal tumor cells, increased RhoE expression correlates with reduced metastatic ability, whereas in melanoma cells, RhoE promotes cell migration and invasion[10,12,25]. To explore the role of RhoE in metastasis of gastric cancer, we knocked down RhoE in the SGC7901-M cell-line, which has a high metastatic potential and we induced its expression in the SGC7901-NM cell-line, which is poor at metastasis. Subsequently, *in vitro* assays demonstrated that increased RhoE promoted cellular motility and invasiveness, while decreased RhoE led to a non-invasive character of gastric cancer cells. These results were further confirmed by the *in vivo* assay. It is well-known that enhanced cancer cell migration and invasion are necessary steps for the final formation of metastases. Therefore, in gastric cancer cells, RhoE might be a functional metastasis-promoting gene. Nevertheless, we also noticed that the cell morphology changed a lot after alteration of RhoE expression. Further investigations found that this morphologic change might be caused by disappearance of stress fiber (Figure S3B), rather than change of cell proliferation. 

As reported, RhoE regulates cancer metastasis, and does so mostly by inhibiting the ROCK/MYPT pathway[26]. This section was investigated in another research study by our group (data not shown). In this study, we applied PCR Array analysis for the identification of other downstream genes of RhoE in gastric cancer metastasis, with the objective of determining the underlying mechanisms of tumor metastasis. Consequently, we obtained 6 differentially expressed metastasis-related genes following up-regulation of RhoE (CXCR4, VEGFA, CTSK, MMP7, CD82 and CTSL1). MMP7 (matrix metalloproteinase-7) belongs to the MMP family of proteins, which are involved in the breakdown of the extracellular matrix in both physical and pathological condition[27]. 

It was reported that elevated expression of MMP7 enhanced the invasive ability of cancer cells[28]. In PCR array analysis, MMP7 was found to be down-regulated in highly invasive SGC7901-NM-RhoE cells, which was contradictory to previous reports. Thus, we considered MMP7 of less importance in our study and did not verify its expression by Western blot. For similar reason, we did not check the expression of CTSL1. To our surprise, among the 4 genes detected by Western blot analysis, only the expression of CXCR4 was found consistent with the result obtained by PCR Array analysis. Further investigation showed that up-regulation of CXCR4 in gastric cancer cells could partially restore the invasive ability, which was suppressed by RhoE knock-out. By contrast, down-regulation of CXCR4 diminished the invasion of gastric cancer cells, which was induced by over-expressed RhoE. Thus, CXCR4 may be a potential down-stream effector of RhoE in gastric cancer metastasis.

CXCR4, together with its ligand CXCL12 (namely the CXCL12 / CXCR4 axis), is well known to be involved in many aspects of tumor progression, especially in tumor metastasis. It was previously reported that the CXCL12 / CXCR4 axis was essential for metastatic cancer cells to disperse to organs and thereby allow tumor cells to access cellular niches that favor tumor-cell survival and growth[29]. Besides that, activated CXCR4 requires binding to G-proteins to transfer extracellular signals into the cell and initiate divergent responses[23]. In addition, when considering that RhoE is a member of the G-protein family, we deemed that RhoE could enlarge the repertoire of CXCL12 / CXCR4 axis signals by increasing the expression of the CXCR4 receptor. By contrast, RhoE might bind to CXCR4 and mediate downstream signal transduction, which might finally result in cancer metastasis. Furthermore, we previously demonstrated that RhoE participates in hypoxia- induced EMT, a key process that contributes to cancer metastasis, in which CXCR4 is also involved. These close connections between RhoE and CXCR4 strongly suggested that RhoE enhanced metastasis by up-regulating CXCR4 in gastric cancer. The detailed mechanisms responsible for these pathways needs further study. 

## Conclusion

Our study demonstrated that RhoE was overexpressed in gastric cancer and correlated with cancer progression. Overexpression of RhoE also correlated with shorter survival time in cancer patients. RhoE might thus function as an effector of invasion and metastasis in gastric cancer by augmenting the expression of CXCR4. Our data provide a new molecular mechanism that might be of assistance in developing an effective treatment for gastric cancer metastasis.

## Supporting Information

Figure S1 **RhoE promoted the migratory and invasive abilities of gastric cancer cell-lines MKN45 and MKN28 *invitro*.** (**A**), The migratory ability of the cells was evaluated with a wound-healing assay. The wound widths of each sample and at different time-points were measured using a phase-contrast microscope (Olympus, Tokyo, Japan), and the closure ratio was calculated in accordance with the following formula: Wound Closure (%) = (width 0 h) – (width 24 h) / width 0 h. *P <0.05. Then these results were then compared to those of the control cells. (**B**), Tumor cell invasion activities were measured by Transwell chamber assay. Representative image fields of invasive cells on the membrane are shown. Data are represented as normalized cellular invasion (invasion index) relative to control cells. *P <0.05. The images shown are representative of three experiments.
(TIF)

Figure S2 **Verification of CXCR4 expression by Western blot.** CXCR4 was up-regulated in SGC7901-M-siRhoE cells after treatment with lentivirus while CXCR4 expression was up-regulated in SGC7901-NM-RhoE cells after treatment with siRNA. CXCR4 protein levels were confirmed by Western blot analysis. β-actin expression was used as an internal control.
(TIF)

Figure S3 **(**A**), Cell growth rate was determined by MTT assay.** Cells were seeded on a 96-well plate at 2 x 103 cells/well in RPMI 1640 containing 10% fetal calf serum. Each sample had three replicates. The medium was replaced at 2-day intervals. Viable cells were counted by the MTT assay within 1st to 7th day. Briefly, cells were incubated with 50 μl of 0.2% MTT for 4 h at 37 °C in the 5% CO2 incubator. Following MTT incubation, cells were lysed in 150 μl of DMSO and the absorbance at 490 nm was obtained using the 96-well plate reader (Thermo, USA). As shown in the figure, the growth rate of SGC7901-M-Control and SGC7901-M-siRNA cells show no difference (p > 0.05), and SGC7901-NM-Control and SGC7901-NM-RhoE show the same result (p > 0.05). (**B**), **cell morphology was observed by confocal laser scanning microscopy**. Briefly, cells seeded on glass coverslips were fixed with 3.7% paraformaldehyde and then permeabilized with Triton X-100. After being blocked with BSA, coverslips were then incubated with primary antibodies of F-actin (1:50, Abcam, USA) in 1% BSA/PBS overnight at 4°C. Coverslips were washed three times with PBS before incubation with appropriate Alexa Fluor-conjugated secondary antibodies (Invitrogen, USA) for 1 h at room temperature. A Zeiss LSM 510 confocal microscope using a × 40/1.3 NA objective was used to photograph samples. As shown in the figure, stress fibers (white arrow) were clearly observed in both SGC7901-M-Control and SGC7901-M-siRhoE cells, which presented with similar cell morphology. Meanwhile, up-regulation of RhoE expression in SGC7901-NM cell significantly reduced the stress fibers and SGC7901-NM-RhoE cells were more spread and extended more extensions than SGC7901-NM-Control cell did.
(TIF)

Table S1 **Multivariate analysis based on Cox’s proportional hazards model.**
(DOC)
