# Supplementary figures and images for: RhoE Promotes Metastasis in Gastric Cancer through a Mechanism Dependent on Enhanced Expression of CXCR4
Source: PLoS One. 2013 Nov 29;8(11):e81709. doi: 10.1371/journal.pone.0081709 (PMC3843694; doi:10.1371/journal.pone.0081709)

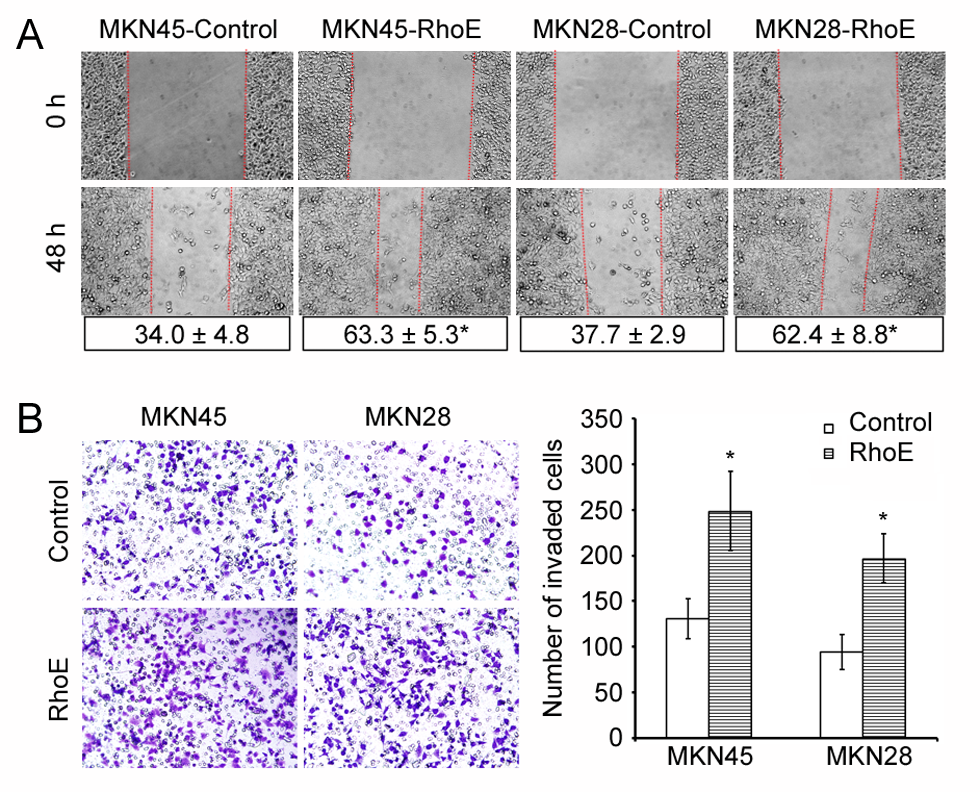

Supplement: Figure S1 — RhoE promoted the migratory and invasive abilities of gastric cancer cell-lines MKN45 and MKN28 invitro. (A), The migratory ability of the cells was evaluated with a wound-healing assay. The wound widths of each sample and at different time-points were measured using a phase-contrast microscope (Olympus, Tokyo, Japan), and the closure ratio was calculated in accordance with the following formula: Wound Closure (%) = (width 0 h) – (width 24 h) / width 0 h. *P <0.05. Then these results were then compared to those of the control cells. (B), Tumor cell invasion activities were measured by Transwell chamber assay. Representative image fields of invasive cells on the membrane are shown. Data are represented as normalized cellular invasion (invasion index) relative to control cells. *P <0.05. The images shown are representative of three experiments. (TIF) [file pone.0081709.s001.tif]

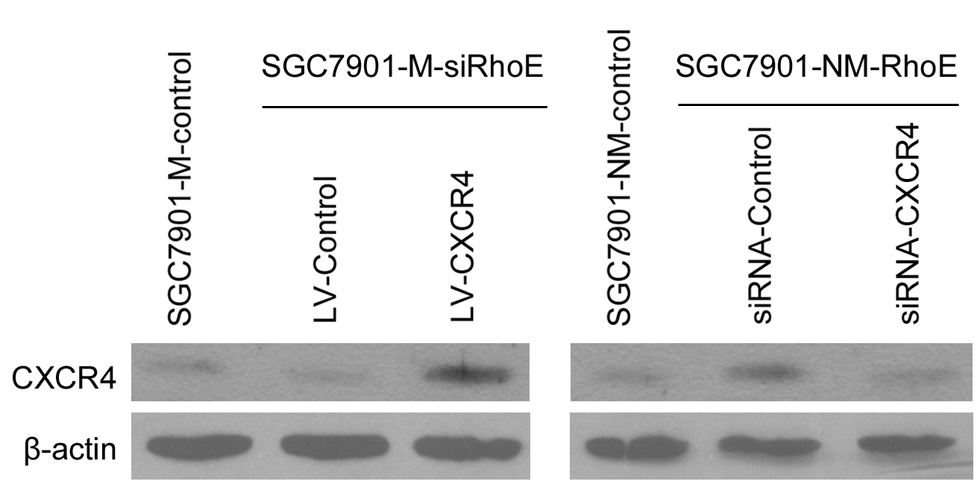

Supplement: Figure S2 — Verification of CXCR4 expression by Western blot. CXCR4 was up-regulated in SGC7901-M-siRhoE cells after treatment with lentivirus while CXCR4 expression was up-regulated in SGC7901-NM-RhoE cells after treatment with siRNA. CXCR4 protein levels were confirmed by Western blot analysis. β-actin expression was used as an internal control. (TIF) [file pone.0081709.s002.tif]

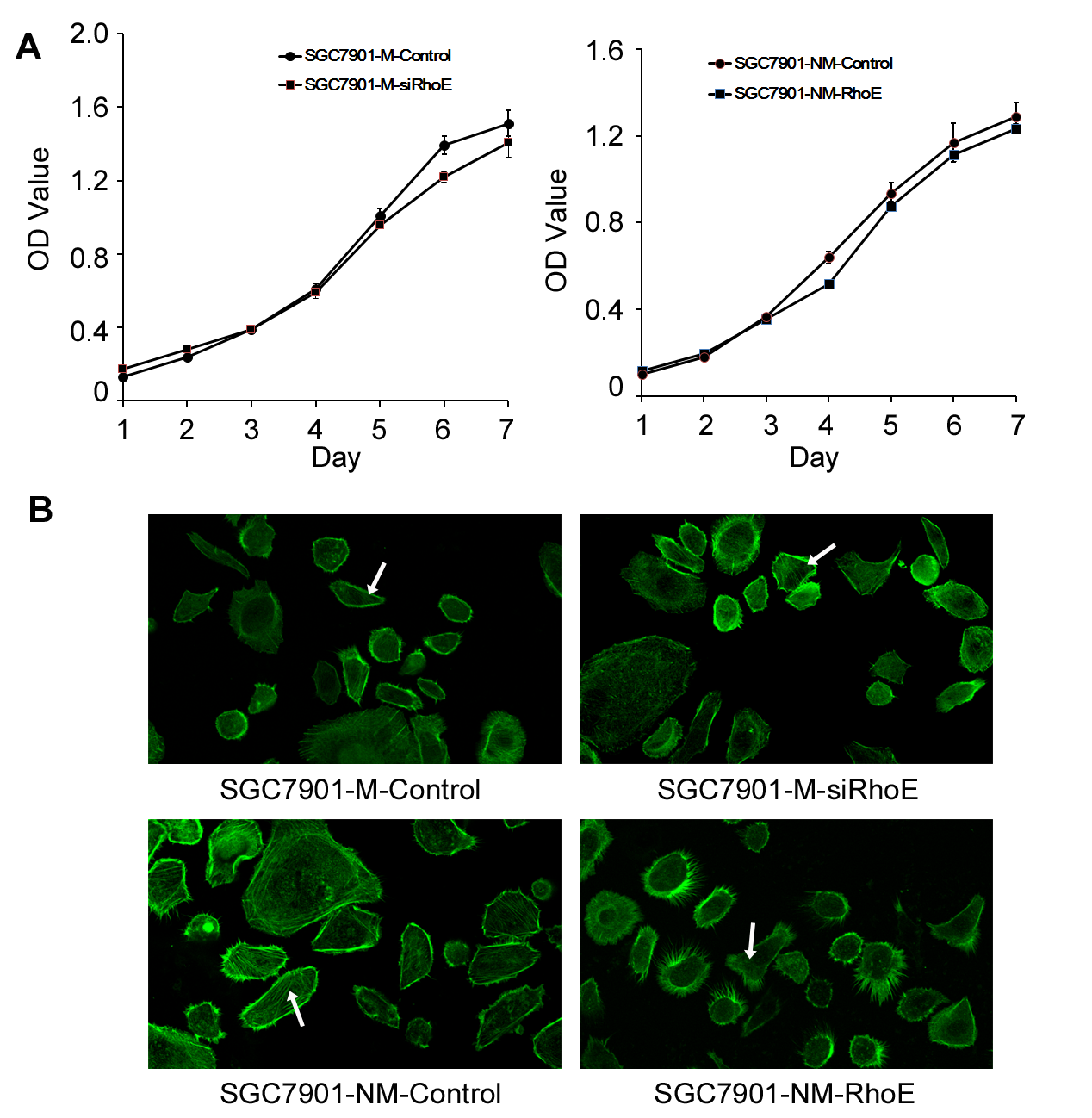

Supplement: Figure S3 — (A), Cell growth rate was determined by MTT assay. Cells were seeded on a 96-well plate at 2 x 10^3^ cells/well in RPMI 1640 containing 10% fetal calf serum. Each sample had three replicates. The medium was replaced at 2-day intervals. Viable cells were counted by the MTT assay within 1st to 7th day. Briefly, cells were incubated with 50 μl of 0.2% MTT for 4 h at 37 °C in the 5% CO2 incubator. Following MTT incubation, cells were lysed in 150 μl of DMSO and the absorbance at 490 nm was obtained using the 96-well plate reader (Thermo, USA). As shown in the figure, the growth rate of SGC7901-M-Control and SGC7901-M-siRNA cells show no difference (p > 0.05), and SGC7901-NM-Control and SGC7901-NM-RhoE show the same result (p > 0.05). (B), cell morphology was observed by confocal laser scanning microscopy. Briefly, cells seeded on glass coverslips were fixed with 3.7% paraformaldehyde and then permeabilized with Triton X-100. After being blocked with BSA, coverslips were then incubated with primary antibodies of F-actin (1:50, Abcam, USA) in 1% BSA/PBS overnight at 4°C. Coverslips were washed three times with PBS before incubation with appropriate Alexa Fluor-conjugated secondary antibodies (Invitrogen, USA) for 1 h at room temperature. A Zeiss LSM 510 confocal microscope using a × 40/1.3 NA objective was used to photograph samples. As shown in the figure, stress fibers (white arrow) were clearly observed in both SGC7901-M-Control and SGC7901-M-siRhoE cells, which presented with similar cell morphology. Meanwhile, up-regulation of RhoE expression in SGC7901-NM cell significantly reduced the stress fibers and SGC7901-NM-RhoE cells were more spread and extended more extensions than SGC7901-NM-Control cell did. (TIF) [file pone.0081709.s003.tif]
