# Supplementary material for: RhoE Promotes Metastasis in Gastric Cancer through a Mechanism Dependent on Enhanced Expression of CXCR4
Source: PLoS One. 2013 Nov 29;8(11):e81709. doi: 10.1371/journal.pone.0081709 (PMC3843694; doi:10.1371/journal.pone.0081709)
Supplement: Table S1 — Multivariate analysis based on Cox’s proportional hazards model. (DOC) [file pone.0081709.s004.doc]

**Supplementary Table. Multivariate analysis based on Cox’s proportional hazards model**

| Risk factors | Hazard Ratio | 95% CI | P |
| --- | --- | --- | --- |
| TNM staging |  |  |  |
| T |  |  |  |
| T1 | 1.00 | - | - |
| T2 | 2.50 | 0.72-8.68 | 0.150 |
| T3 | 2.39 | 0.65-8.69 | 0.188 |
| T4 | 7.97 | 1.96-32.39 | 0.004 |
| N |  |  |  |
| N0 | 1.00 | - | - |
| N1 | 2.72 | 1.20-6.21 | 0.017 |
| M |  |  |  |
| M0 | 1.00 | - | - |
| M1 | 6.39 | 2.90-14.05 | <0.001 |
| RhoE |  |  |  |
| - | 1.00 | - | - |
| + | 2.10 | 0.75-5.89 | 0.16 |
| ++ | 7.16 | 2.57-19.95 | <0.001 |
| +++ | 7.73 | 2.45-24.40 | <0.001 |
